# Changes in the C-terminal, N-terminal, and histidine regions of amelogenin reveal the role of oligomer quaternary structure on adsorption and hydroxyapatite mineralization

**DOI:** 10.3389/fphys.2022.1034662

**Published:** 2022-11-29

**Authors:** Jinhui Tao, Emma Hanson, Alice C. Dohnalkova, Garry W. Buchko, Biao Jin, Wendy J. Shaw, Barbara J. Tarasevich

**Affiliations:** ^1^ Pacific Northwest National Laboratory, Richland, WA, United States; ^2^ School of Molecular Biosciences, Washington State University, Pullman, WA, United States

**Keywords:** amelogenin, oligomer, atomic force microscopy, hydroxyapatite, amorphous calcium phosphate

## Abstract

Adsorption interactions between amelogenin and calcium phosphate minerals are believed to be important to amelogenin’s function in enamel formation, however, the role of specific amino acid residues and domains within the protein in controlling adsorption is not well known. We synthesized “mechanistic probes” by systematically removing charged regions of amelogenin in order to elucidate their roles. The probes included amelogenin without the charged residues in the N-terminus (SEKR), without two, three, or eight histidines (H) in the central protein region (H2, H3, H8), or without the C-terminal residues (Delta). *In-situ* atomic force microscopy (AFM) adsorption studies onto hydroxyapatite (HAP) single crystals confirmed that the C-terminus was the dominant domain in promoting adsorption. We propose that subtle changes in protein-protein interactions for proteins with histidines and N-terminal residues removed resulted in changes in the oligomer quaternary size and structure that also affected protein adsorption. HAP mineralization studies revealed that the oligomer-HAP binding energy and protein layer thickness were factors in controlling the amorphous calcium phosphate (ACP) to HAP induction time. Our studies with mechanistic probes reveal the importance of the oligomer quaternary structure in controlling amelogenin adsorption and HAP mineralization.

## Introduction

The biomineralization of hard tissue structures including mollusk shells, vertebrate teeth, and vertebrate bones involves a complex interplay between proteins, mineral ions, and mineral precipitates (Lowenstam and Weiner, 1989). Proteins and polysaccharides are excreted into extracellular spaces that mineralize over time. The extracellular biomacromolecules are believed to guide the mineralization process by promoting nucleation, affecting growth, and controlling phase transformations of metastable precursors to the final minerals (Simmer and Fincham, 1995; Mann, 2001). In some cases, growth involves particle mediated processes where proteins promote the aggregation and phase transformation of precursor particles into the final mineral shape and phase (Jin et al., 2018). The protein matrices either remain in place as part of the hard tissue structure, such as collagen matrices in bone or tooth dentin (George and Veis, 2008), or become almost completely removed by enzymes, such as in tooth enamel (Bartlett, 2013).

Amelogenin is a protein that is critical to the formation of HAP in tooth enamel (Fincham et al., 1999). The absence of amelogenin in knock-out mice results in greatly reduced HAP thickness and HAP that is lacking in mesoscale order (Hu et al., 2016). Ameloblasts secrete amelogenin as well as other proteins into the extracellular matrix during the secretory phase of enamel development (Fincham and Simmer, 1997). It was suggested by diffraction studies that ACP is initially formed within the matrix (Beniash et al., 2009) and ACP particles assemble into long thin structures that are parallel to each other and separated by protein matrix (Simmer et al., 2012). ACP transforms to HAP in the later secretory stages (Beniash et al., 2009) and then HAP crystals further widen and thicken during the maturation phase (Simmer et al., 2012; Bartlett, 2013). The adsorption of amelogenin onto calcium phosphate is believed to be critical in affecting each step *in vivo*, promoting the alignment of ACP particles into ribbons, inhibiting the transformation of ACP to HAP until protein is removed by the protease MMP20 (Beniash et al., 2009; Shin et al., 2020; Bartlett et al., 2021), and inhibiting the lateral growth of HAP crystals until protein is removed by the enzyme KLK4 (Simmer et al., 2012; Bartlett, 2013). Numerous *in vitro* studies have shown the importance of amelogenin adsorption onto calcium phosphates in affecting the ACP to HAP transformation and crystal growth (Kwak et al., 2009; Kwak et al., 2016; Tao et al., 2019).

Although the adsorption of amelogenin onto calcium phosphates appears to be critical to its function in enamel formation, there are questions as to what primary amino acid sequences or domains in amelogenin control adsorption. Amelogenin is less charged than other biomineralization proteins and self-assembles into oligomers that in turn assemble into larger nanospheres (Fang et al., 2011). The quaternary structure of the oligomers, defined as the spatial arrangement of monomers and the size of the oligomers, has been found to be very unique, consisting of pairs of dimers that assemble to form a ring (Fang et al., 2011). Although there was a distribution of ring diameters, the most prominent ring consisted of six pairs of dimers to form a dodecamer. It has been suggested that the C-terminal domain (residues A167—D180) (Moradian-Oldak et al., 2002) and the N-terminal domain (residues M1–M42) (Aoba et al., 1989; Lu J. X. et al., 2013) may have roles in the adsorption of amelogenin since all of the 13 charged residues in the protein’s primary sequence are located in these regions. These regions have been found to be located on the outside of amelogenin assemblies (Moradian-Oldak et al., 2001) and may interact directly with mineral surfaces. In addition to the highly charged residues, phage display techniques have suggested that the weakly basic histidines found in the central region of amelogenin may also be important for binding onto calcium phosphate (Gungormus et al., 2008; Gungormus et al., 2012). There is also evidence that the N-terminus and histidine residues have roles in promoting protein-protein interactions involved in oligomer and nanosphere self-assembly (Paine and Snead, 1997; Moradian-Oldak et al., 2000; Bromley et al., 2011).

In this study, we set out to test the role of polar/charged N-terminal, C-terminal, and histidine amino acid residues of amelogenin in modulating adsorption interactions with HAP and the ACP to HAP transformation during mineralization by selectively removing the residues from amelogenin. This approach is complimentary to examining the role of specific amino acid residues by studying smaller peptides containing those residues. The resulting “mechanistic probes” consisted of amelogenin with four amino acids removed in the N-terminus (SEKR), two to eight histidine residues removed in the central region (H2, H3, H8), and the C-terminus removed (Delta). We studied the adsorption of the wild-type protein, rpM179, and the mechanistic probes onto HAP single crystals using *in situ* AFM and the kinetics of mineralization of HAP in the presence of the variants. Our studies confirm that the C-terminus is the most important domain involved in amelogenin adsorption, consistent with other studies (Moradian-Oldak et al., 2002). We also conclude that the removal of residues in the N-terminus and histidines in the central region can change the quaternary structure of the adsorbate oligomers that in turn affect their adsorption interactions.

## Materials and methods

### Amelogenin preparation and purification

**FIGURE 1:**
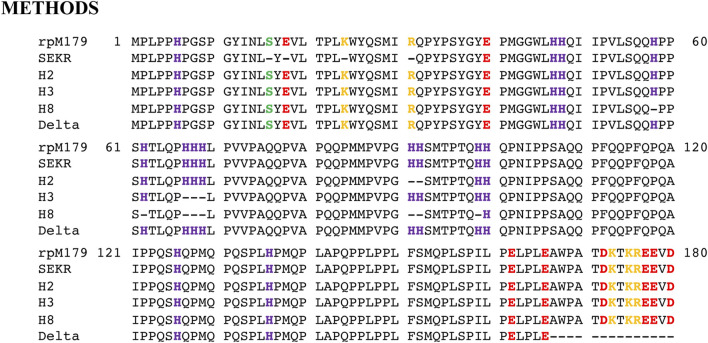
Primary amino acid sequences for rpM179 and the mechanistic probes SEKR, H2, H3, H8, and Delta. The amino acids that are removed from rpM179 to form the mechanistic probes are indicated by dashes. SEKR is formed by the removal of S16, E18, K24, and R31 in the N-terminus, H2 by the removal of H91 and H92, H3 by the removal of H67, H68, and H69, H8 by the removal of H58, H62, H67, H68, H69, H91, H92, and H99, and Delta by the removal of A167WPATDKTKREEVD180 in the C-terminus. Amino acids with side chains that are acidic, strongly basic, and weakly basic (histidines) are colored red, yellow, and purple, respectively. The serine at S16 that undergoes post translational phosphorylation is colored green. Note that the methionine at M1 is removed during the recombinant synthesis process.

Synthetic genes for the five mechanistic probe variants, SEKR, H2, H3, H8, and Delta, were prepared commercially (DNA2.0, Menlo Park, CA) by making the appropriate variations to the original *E. coli* optimized rpM179 DNA sequence and inserting into the expression vector pJexpress414. These plasmids were used to transform BL21 (DE3) competent cells (Novagen, Madison, WI) *via* a heat-shock method. Starting with frozen 12% glycerol stocks prepared from a single colony, uniformly ^15^N-labelled rpM179 and the five protein variants were expressed using a minimal media based autoinduction protocol (Studier, 2005). Because the expressed protein contained no affinity tag, it was purified following a previously described protocol starting by lysing the frozen cell pellet at 70°C in 40 ml of 2% acetic acid over 30 min (Buchko et al., 2013). The entire solution was then dialyzed twice in 5 L of 2% acetic acid (3.5 kDa MW cutoff), centrifuged, and the supernatant applied onto an Xbridge Preparative C18 (5 μm, 10 mm × 250 mm) reverse phase column (Water, Milford, MA). After application of a gradient of 70% aqueous CH_3_CN in 0.05% TFA, the major fractions containing amelogenin were pooled, frozen at −80°C, lyophilized, and stored at 4°C until ready to use. The primary amino acid sequence for the non-native amelogenin variants were verified by the collection of an ^1^H-^15^N HSQC spectrum in 2% acetic acid and comparison to the assigned ^1^H-^15^N HSQC spectrum for full length rpM179 (Buchko et al., 2008a). The spectra were essentially identical except for positions at, or 1–2 residues from, the site of the amino deletions (Buchko et al., 2013). Intact protein mass spectra for each of the constructs (unlabeled protein) further confirmed the primary amino acid sequence of the expressed protein as the major m/z peak for each of the protein constructs corresponded to its expected molecular weight. Figure 1 shows the complete sequences of each protein.

### Protein solution preparation

Solutions of rpM179 and the variants were prepared by dissolving 5–10 mg/ml of protein in water. After one to three days of dissolution, the solutions were filtered with 0.2 µm pore membranes, the concentrations of the protein solutions were determined using UV-vis spectroscopy (Nanoprobe), and the stock protein solutions were diluted into 25 mM Tris-HCl buffer adjusted to pH 8.0. These solutions were used for the dynamic light scattering (DLS) and AFM studies. The pH 8.0 solution condition was used in order to be higher than the isoelectric point of the proteins to avoid extensive protein agglomeration. The highest isoelectric point is for Delta at 7.23 as shown in Supplementary Table S1. Also, amelogenin nanospheres have narrow size distributions at this pH (Moradian-Oldak et al., 1998). This pH is in within the range of pH values up to pH 8.5 found within enamel tissue (Lyman and Waddell, 1977; Lacruz et al., 2010). The net charge and hydrophobicity of the proteins were calculated using the protein calculator at http://protcalc.sourceforge.net/ and ProtParam tool at http://web.expasy.org/protparam/ (Supplementary Figure S8 and Supplementary Table S1).

### Dynamic light scattering

DLS measurements of the protein solutions were performed using a Brookhaven Instruments 90 Plus equipped with a 657 nm wavelength, 35 mW laser. Time dependent fluctuations in the scattered intensity were measured using a Bl-APD digital correlator. Protein solutions were analyzed using a 90° scattering angle at 25.0 °C. Data were collected as co-added runs of 2 min collected for a total of 6–12 min. The autocorrelation functions were deconvoluted to obtain size distributions using the non-negatively constrained least squares fit (multiple pass NNLS). The nanosphere size was obtained as the most prominent peak in the volume-weighted distribution and was averaged over at least 3 runs. The standard deviation of the mean nanosphere size averaged 12.2% over all measurements.

### Atomic force microscopy imaging

Details of the use of AFM to study amelogenin adsorption onto the (100) face of micron-sized single crystals of HAP were described in our previous publications (Tao et al., 2015; Tao et al., 2019) and in the Supplementary Information. The adsorption kinetics of proteins in 25 mM Tris buffer, pH 8.0, ambient temperature solutions at concentrations of 15.6 μg/ml and 125 μg/ml were studied. Adsorption amounts were obtained from the coverage of adsorbates determined from the adsorbate areas calculated by the Particle and Pore Analysis module included in the SPIP 5.1.4 software. The adsorption kinetics of rpM179 and H8 were also studied at 7.8, 31.3, 62.5, 180 and 250 μg/ml (ppm) protein concentration in order to have enough data points to perform Hill analyses of the equilibrium adsorption coverages as a function of protein concentration, as described in detail previously (Tao et al., 2015; Tao et al., 2019) and in the Supplementary Information. The equilibrium coverages are the coverages obtained from the kinetic curves at maximum adsorption. Equilibrium coverage data of rpM179 from a previous publication (Tao et al., 2019) was included with the newer rpM179 data obtained in this study for the Hill analyses. The equilibrium adsorption coverages for each concentration of wild-type and variant protein were averaged from at least three experiments resulting in standard deviations of 10% or less. Height distributions of the adsorbates from protein solutions at 15.6 μg/ml were determined using the Particle & Pore Analysis module included in the SPIP 5.1.4 software for over 100 adsorbates and the mean of the distribution was determined.

### Transmission electron microscopy studies of protein oligomer structure

The size and structure of oligomers of rpM179, H3, and H8 were studied by placing drops of pH 8, 25 mM Tris•HCl, 62.5 μg/ml or 125 μg/ml protein onto formvar-carbon coated nickel TEM grids placed over an ice bath. The grids were cleaned by plasma exposure immediately before use. After 30–60 s of adsorption time, the grids were blotted, rinsed by placing the grids on top of water droplets, and blotted. NanoW negative stain (5 μl, 2% organotungsten compound, Nanoprobes, Inc.) was dropped onto the grids for 10 s and then blotted and the grids were dried. TEM imaging was performed using a Tecnai T-12 TEM (FEI) with a LaB_6_ filament operating at 120 kV with images obtained in focus or underfocus. Images were collected digitally using an Ultrascan 1000 CCD. Size distributions of oligomer diameters were obtained by the measurement of 100–300 oligomers.

### Mineralization studies

The mineralization of calcium phosphate was studied in the presence of rpM179 and the variants. Stock solutions of protein were dissolved in water as described above and were diluted into water to give final concentrations of 200 μg/ml protein. Aliquots of 50 mM CaCl_2_ and 30 mM KH_2_PO_4_ stock solutions in water were sequentially added to give final concentrations of 2.5 mM CaCl_2_ and 1.5 mM KH_2_PO_4_. The pH was increased to a final pH of 7.8 by slowly adding KOH. The sample vial was placed into a jacketed beaker at 22°C and the growth kinetics were monitored by changes in pH over a 6 h time period using a Microelectronics combination electrode and Orion pH meter, a method commonly used to monitor HAP formation (Kwak et al., 2009). Induction times for ACP to HAP transformation were obtained from the pH change curves. Reported mean induction times were obtained over three to six experimental repetitions.

### Transmission electron microscopy studies of mineral structure

Samples for TEM analysis of mineral were prepared by dropping mineralization suspensions after 0.5 and 24 h onto formvar-carbon coated copper grids. TEM imaging was performed using a Tecnai T-12 TEM (FEI) with a LaB_6_ filament operating at 120 kV. Images were collected digitally using an Ultrascan 1000 CCD.

### Statistical Analysis

T-tests at 0.05 significance level were used to determine the statistical significance of AFM determined height differences, TEM determined diameter differences, and induction time differences between the variants and rpM179.

## Results

### Adsorption of mechanistic probes onto the HAP (100) surface

**FIGURE 2:**
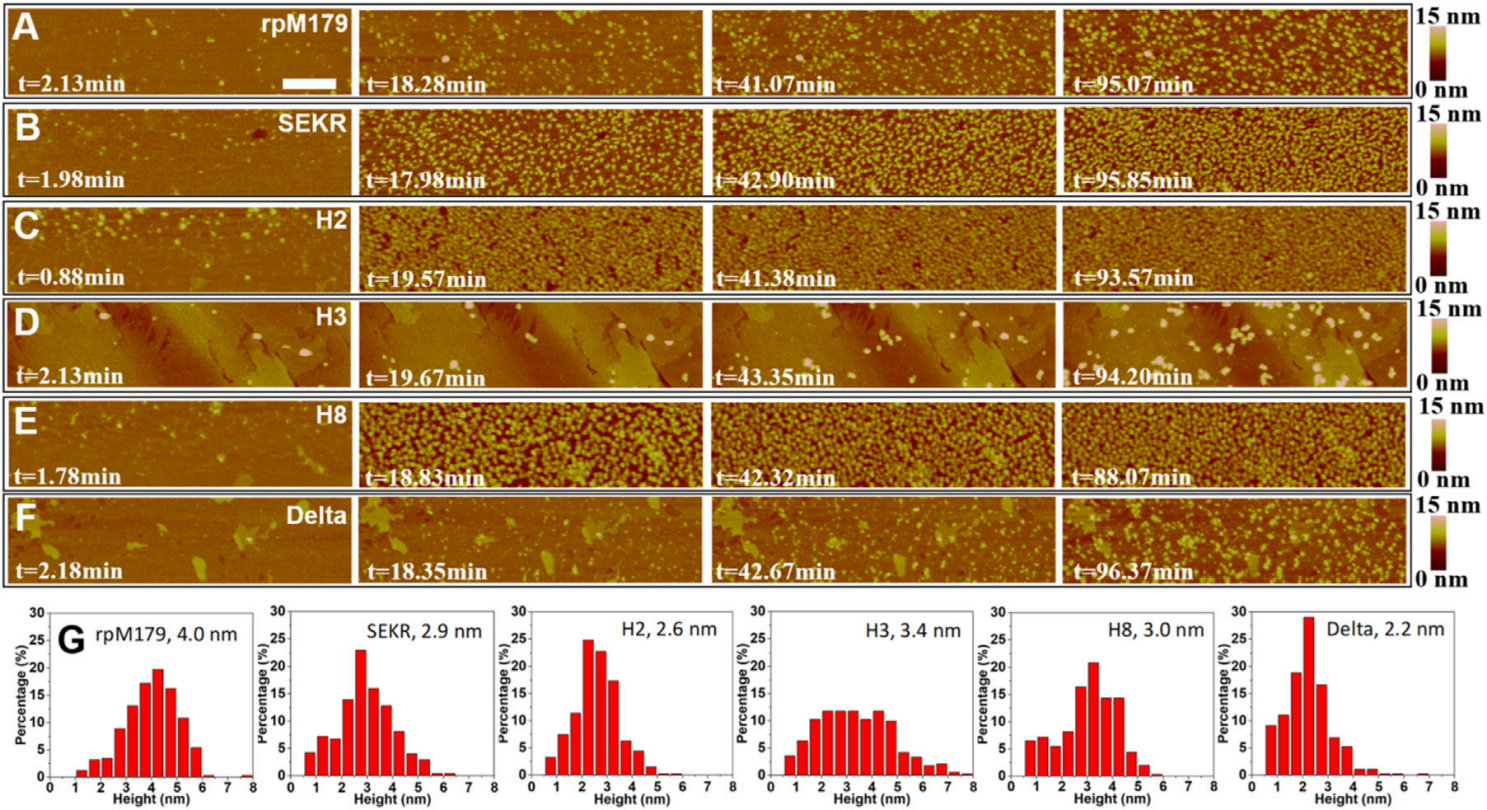
**(A–F)** *In situ* AFM images of adsorbates from 15.6 μg/ml protein, 25 mM Tris•HCl, pH 8 solutions at various time periods and showing at most one monolayer of adsorbates. The zero point of time is defined as the time when protein solutions are injected into the AFM liquid cell. The scale bar is 200 nm. All the micrographs are the same size. **(G)** Height distributions of the adsorbates with the mean height shown in the label. The second and third images in **(A)** were taken from Tao et al., 2019.

The adsorption of mechanistic probes and wild-type rpM179 onto single crystal HAP (100) surfaces was studied by *in situ* AFM. Figures 2A–F and Supplementary Videos S1–S6 show AFM images of the adsorbates from solutions at various time points and a protein concentration of 15.6 μg/ml relative to the bare HAP substrates shown in Supplementary Figure S2. Individual adsorbates could be resolved at this low concentration at early time points and height distributions were obtained as shown in Figure 2G. The mechanistic probe adsorbates were 2–3 nm in height, statistically smaller than the rpM179 adsorbates at 4–5 nm. The protein adsorbates were smaller than the larger nanospheres that were detected in solution by DLS (Supplementary Figure S1). We call these adsorbates “oligomers” and define them as smaller subunits of the larger nanospheres (Fang et al., 2011). We propose that the oligomers adsorb onto the surface by the disassembly of the nanospheres present in solution as we have shown previously (Tarasevich et al., 2009; Tao et al., 2015) and/or the oligomers are present in solution although not detected by DLS and preferentially adsorb onto the surface.

**FIGURE 3:**
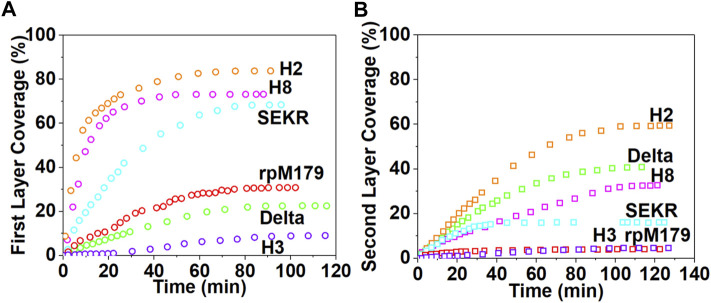
The kinetics of protein adsorption determined from the areal coverage of oligomer adsorbates obtained from AFM data for **(A)** the first layer of adsorption from solutions at 15.6 μg/ml and **(B)** the second layer of adsorption from solutions at 125 μg/ml. The data for rpM179 is from Tao et al., 2019.

The images in Figures 2A–F show that at low coverage, the adsorbates consisted of isolated oligomers or small clusters of oligomers, and at high coverage, the adsorbates formed dense monolayers of oligomers. The adsorbates for H3 and Delta appeared to consist of wider assemblies of oligomers, suggesting different interactions with the HAP surface compared to rpM179. The protein coverage was determined from the AFM-measured area of the oligomer, adjusted for tip diameter, relative to the entire area. Figure 3A shows the kinetics of adsorption coverage as a function of time at 15.6 μg/ml and indicated that the protein coverage increased with time until an equilibrium coverage was reached. H3 and Delta resulted in the lowest equilibrium protein coverages at 15.6 μg/ml, and SEKR, H2, and H8 had the highest coverages.

Adsorption studies at higher protein concentrations at 125 μg/ml (Supplementary Figures S3, S4, and Supplementary Videos S7–S12) showed that adsorption at first resulted in an almost complete monolayer of oligomers followed by the adsorption of a second layer of oligomers. Figure 3B shows the equilibrium adsorption coverage of the second layer as a function of time at 125 μg/ml. There was a small amount of second layer adsorption for rpM179 and H3 and a larger amount of second layer adsorption for SEKR, H2, H8, and Delta. Supplementary Figure S4 shows the distribution of second layer oligomers at various time points for rpM179 and H8. The second layer adsorbates of rpM179 were smaller in size than the first layer adsorbates and the second layer adsorbates of H8 changed in size over time as smaller initial adsorbates aggregated to form larger adsorbates.

Adsorption was studied at a wider range of protein concentrations for rpM179 and H8 (Supplementary Videos S5, S11, S13–S15). The equilibrium adsorption amounts at various concentrations were analyzed using the Hill equation that included a cooperative effect on binding quantified by the Hill coefficient, *n*, where protein binding to the surface is enhanced by existing protein adsorbates when *n* is greater than 1 (see Supplementary Information text). Hill plots are shown in Supplementary Figure S5A for an analysis of the first layer of adsorption to obtain oligomer-HAP binding energies and in Supplementary Figure S5B for an analysis of the second layer adsorption to obtain oligomer-oligomer binding energies. Table 1 shows the resulting Hill coefficients, *n*, oligomer-HAP, and oligomer-oligomer binding energies. The data show that the oligomer-HAP binding energy and oligomer-oligomer binding energy were higher for H8 compared to rpM179.

**TABLE 1:** Hill coefficients (*n*) and binding energies (*ΔG*) for oligomer-HAP and oligomer-oligomer interactions on the HAP surface obtained from the first layer adsorption data and the second layer adsorption data, respectively. The adsorption data for rpM179 was new data combined with data from previous publications (Tao et al., 2015; Tao et al., 2019).

Interaction	*n*	Δ*G* (*k*_*B*_ *T*)
rpM179-HAP (100)	3.34 ± 0.40	−17.2 ± 2.9
H8-HAP (100)	0.51 ± 0.06	−19.6 ± 3.3
rpM179-rpM179	0.42 ± 0.01	−7.8 ± 0.4
H8-H8	2.11 ± 0.06	−15.1 ± 0.6

### Oligomer quaternary structure by transmission electron microscopy

**FIGURE 4:**
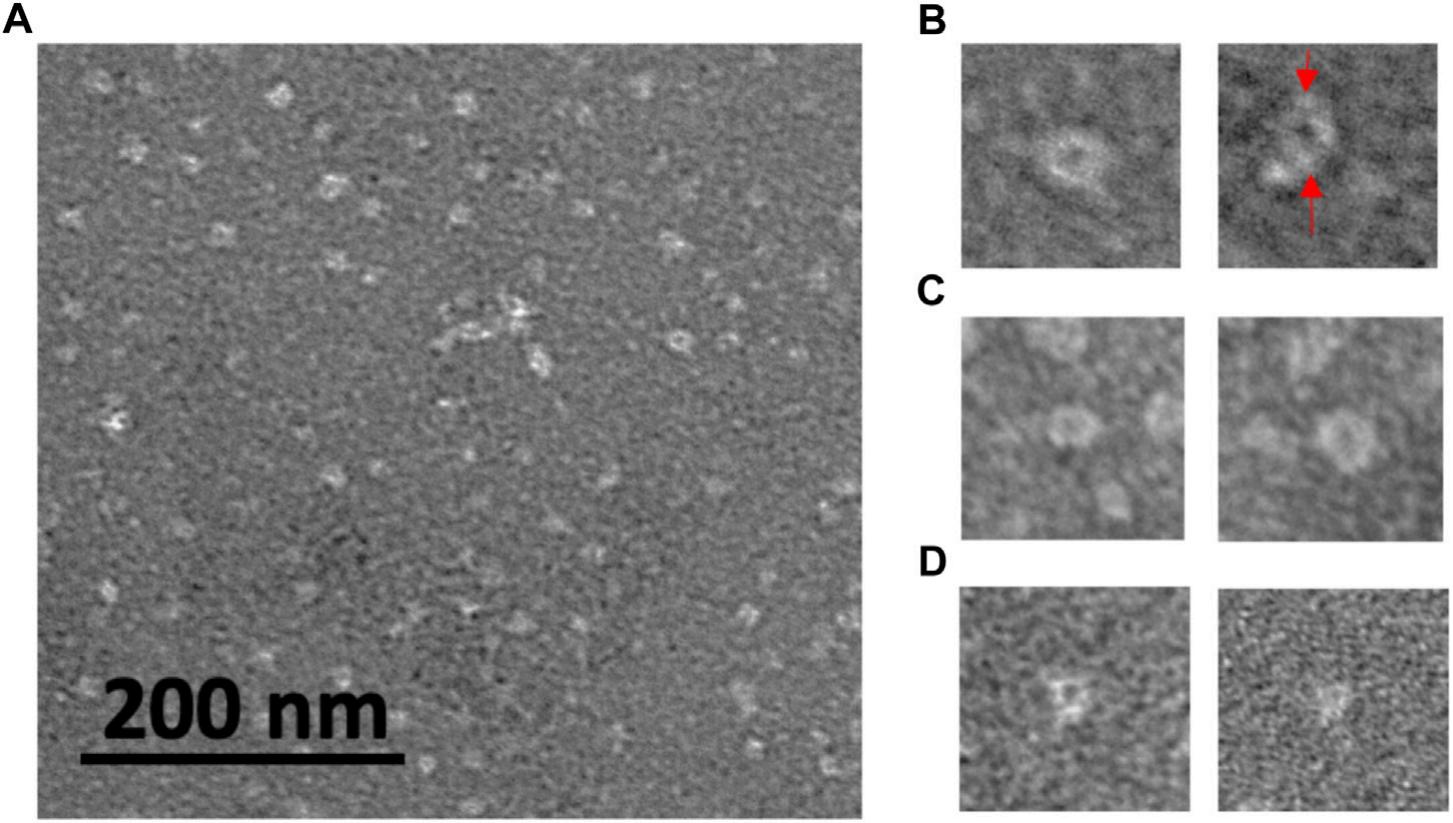
TEM images of protein oligomers adsorbed onto grids for **(A)** rpM179; select images of oligomer rings for **(B)** rpM179 (arrows point to individual dimers), **(C)** H3, and **(D)** H8. The widths of the frames in **(B–D)** are 50 nm.

Further information on the oligomer size and structure was obtained by TEM examination of oligomers adsorbed onto grids. TEM imaging can have higher resolution than AFM imaging and can provide more detail on the oligomer structure. Also, size measurements give oligomer diameters, complimentary information to the oligomer heights obtained by AFM. Figure 4A shows white, unstained oligomeric adsorbates of rpM179 distributed onto the darker, stained grid surface. In some cases, structures consisting of a white ring surrounding a darker center could be resolved (Figure 4B), similar to the ring-like structures previously observed by cryo-EM (Fang et al., 2011). The rings are believed to be formed by the interaction of two monomers to form dimers joined by the C-terminus and the assembly of pairs of dimers into the ring-like structure. Individual dimers were resolved in one of the TEM images in Figure 4B. Ring-like structures were also observed for H3 (Figure 4C) and H8 (Figure 4D). The hole in the ring was not always observed. The ability to see the change in electron density in the center of the ring seemed to be promoted by optimizing the focus conditions and staining. Also, the ability to see the rings would depend on having the orientation of the ring parallel to the surface.

The sizes distributions of the oligomers were determined as shown in Supplementary Figure S6 and the mean diameters of the oligomers were 12.7, 8.9, and 8.6 nm for the rpM179, H3, and H8 proteins, respectively. T-tests showed that the H3 and H8 diameters were significantly smaller than the rpM179 diameter.

### Hydroxyapatite mineralization

**FIGURE 5:**
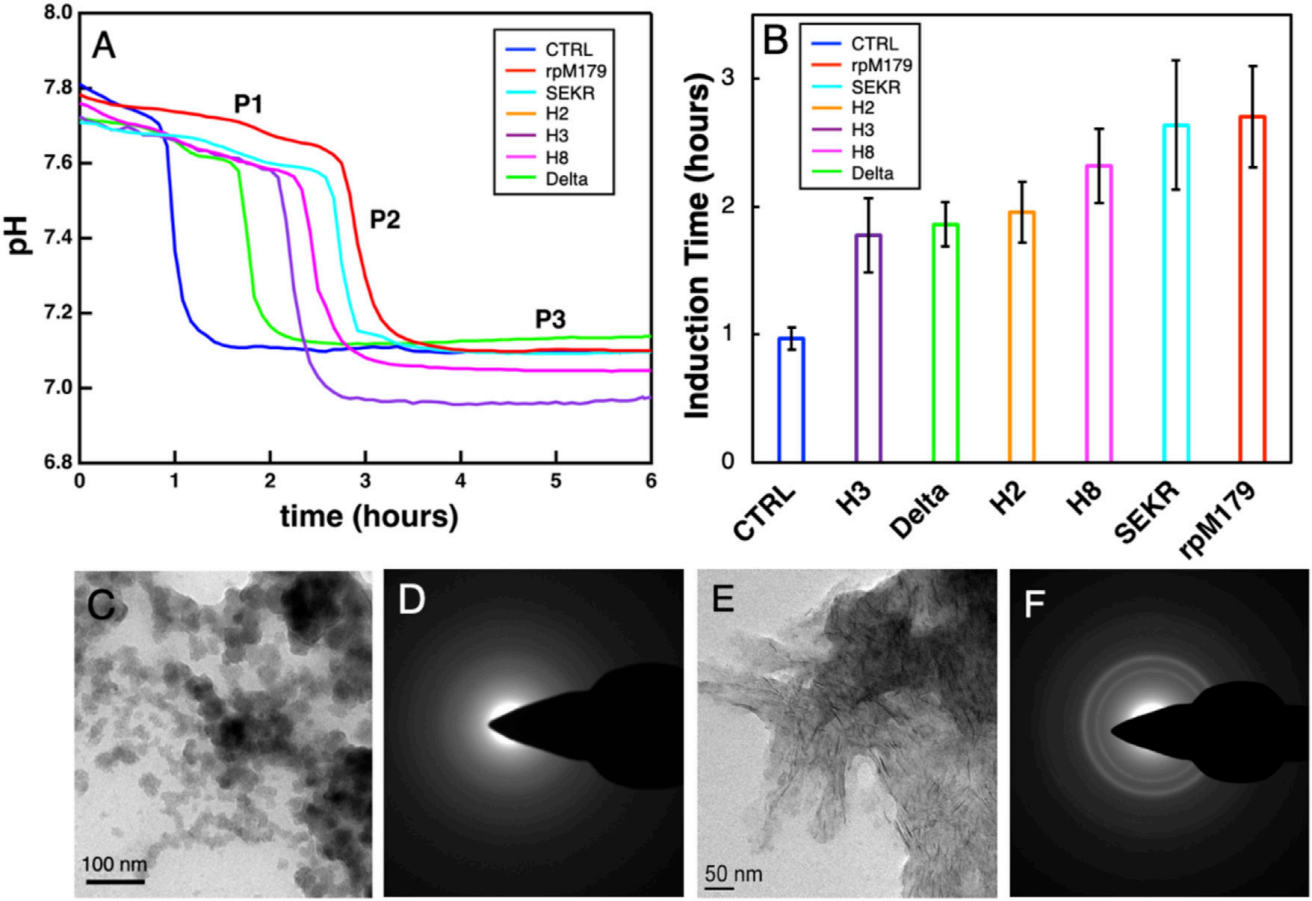
**(A)** Typical curves for the kinetics of mineralization of calcium phosphate in the absence of protein (CTRL) or the presence of 200 μg/ml wild-type and mechanistic probe proteins at 22°C. The rpM179 curve (red) is labeled to show the different stages of mineralization: P1, the formation of ACP; P2, the ACP to HAP transition region; and P3, the growth and ripening of HAP. **(B)** Mean induction times (duration of P1) for the ACP to HAP phase transformation listed from the shortest induction time to the longest induction time. **(C)** TEM image showing ACP nanoparticles formed during P1 (0.5 h) for rpM179. **(D)** TEM electron diffraction pattern for the image in **(C)**. **(E)** TEM image showing HAP ribbons formed at 24 h for rpM179. **(F)** TEM electron diffraction pattern for the image in **(C)**.

The mineralization of HAP with no protein and in the presence of the various proteins at 200 μg/ml was studied by monitoring changes in pH of the mineralization solutions as shown in Figure 5A. There was an initial stage of a slow decrease in pH (P1), followed by a region of a faster decrease in pH (P2), and a third stage of no change in pH (P3). Calcium phosphate nanoparticles were formed at 0.5 h in the P1 region for rpM179 as shown in Figure 5C and the particles were found to be amorphous ACP (Figure 5D). By the P3 region, the calcium phosphate was found to consist of ribbon-like, moderately crystalline HAP imbedded in a low contrast matrix as shown in the image in Figure 5E and diffraction patterns in Figure 5F for rpM179 at 24 h. The other proteins resulted in similar mineral structures at 24 h as shown in Supplementary Figure S7. P2, therefore, was found to correspond to the phase transformation of ACP into HAP and the duration of P1 is the induction time for the ACP to HAP transformation (Ding et al., 2014). Figure 5B shows the mean induction times for the ACP to HAP transformation for the control (CTRL) in the absence of protein, rpM179, and the mechanistic probes. The induction times for the proteins were significantly longer than for the control solution suggesting that the proteins inhibited the ACP to HAP phase transformation. H2, H3, and Delta had significantly shorter induction times compared to the wild-type rpM179.

## Discussion

### Adsorbed oligomer quaternary structure

Amino acid residues thought to be important for the function of amelogenin including the charged C-terminus, histidine residues at various locations, and polar/charged residues in the N-terminus were systematically removed from the protein (Figure 1). Adsorption studies by AFM showed that all of the proteins adsorbed as oligomeric structures. Previous cryo-EM studies found that rpM179 monomers at pH 8 were around 1.8 nm in diameter and aggregated to form dimers and then oligomer rings with the C-terminal domains located on the outside of the oligomers (Fang et al., 2011). The most prominent oligomer in that study was a dodecamer composed of a ring of six dimers. Our TEM studies confirmed that the rpM179 oligomers were shaped like rings.

In contrast to rpM179, the previous cryo-EM studies showed that rpM166 (Delta in our studies) went from monomers to nanospheres with no intermediate oligomeric structure (Fang et al., 2011). Our studies showed that Delta adsorbed as 2.2 nm height oligomers. The Fang et al. study suggests that the Delta adsorbates would not be as highly structured as the rpM179 oligomers. That study indicated that the C-terminal domain is important in forming the highly structured oligomer ring. Recent solid state NMR (ssNMR) studies have shown that the C-terminal domains overlap in an antiparallel conformation to form the dimer (Arachchige et al., 2018). We hypothesized that rpM179 and the variants SEKR, H2, H3, and H8 would form ring-like oligomers because they all contain the C-terminus. The oligomers with C-termini we have studied by TEM (rpM179, H3, and H8) all formed ring-like oligomers, consistent with this hypothesis.

H2, H3, H8, and SEKR all adsorbed as oligomers that were primarily around 2.6–3.0 nm in height, smaller than the rpM179 oligomers. TEM studies confirmed that the oligomer diameters for H3 and H8 were smaller than the rpM179 diameters. The smaller sizes suggest that the quaternary structure of the oligomers has changed with fewer dimers involved in the formation of the ring-like structures found for H3 and H8 compared to rpM179. These variants, therefore, would be smaller than the 12-mer found for rpM179. This indicates that the histidines deleted from H2, H3, and H8, and the S16, E18, K24, and R31 residues deleted from SEKR have a role in promoting the aggregation of protein dimers into oligomeric intermediates. Previously, the N-terminus has been suggested to be important in the self-assembly of amelogenin monomers to form oligomers. For example, DLS studies showed that amelogenin with the N-terminus from 1 to 42 removed resulted in a significant population of amelogenin monomers within the solution (Moradian-Oldak et al., 2000). A yeast two-hybrid assay revealed that a peptide consisting of the N-terminus interacts strongly with amelogenin (Paine and Snead, 1997) and solution NMR studies showed that amelogenin monomers interact first at the N-terminus upon addition of salt (Buchko et al., 2008b). Histidines are believed to promote the aggregation of oligomers to form nanospheres as the pH is increased from 5 to 7.4 because of the reduction in histidine charge (Bromley et al., 2011). Protein-protein interactions can also be promoted by π−π stacking between aromatic rings of histidines (Heyda et al., 2010; Tao et al., 2015). Removal of the SEKR or H residues, therefore, resulted in smaller oligomers because of a reduction in protein-protein interactions.

### Adsorption amounts

The AFM studies showed that there were significant differences in the adsorption amounts for the mechanistic probes and rpM179 onto the HAP (100) face at 15.6 μg/ml. The mechanistic probe protein lacking the C-terminus, Delta, had low amounts of adsorption while all of the other proteins that had a C-terminus, except for H3, had higher protein amounts. This result confirms the importance of the C-terminus in amelogenin binding to HAP resulting in interactions between the C-terminal dimers located on the outside of the oligomers (Fang et al., 2011) and the surface (Moradian-Oldak et al., 2002).

**FIGURE 6:**
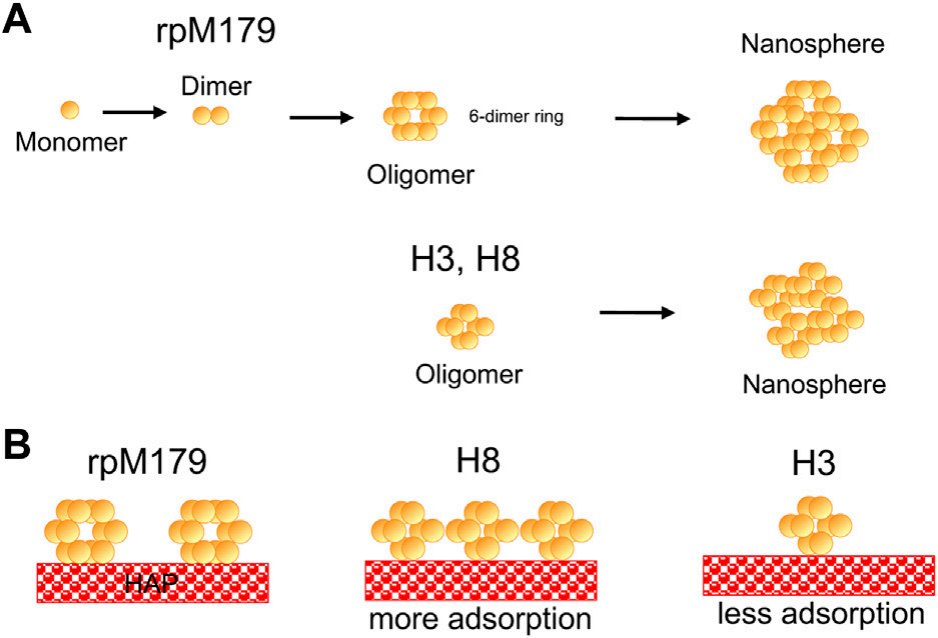
Schematic drawings of **(A)** the hierarchical quaternary structure of rpM179 from monomer to dimer to six-dimer oligomer ring to nanosphere as suggested by the literature (Bromley et al., 2011; Fang et al., 2011) and from oligomer ring to nanosphere for H3 and H8 as suggested by the adsorption and DLS results of this study. Smaller oligomers are shown for H3 and H8 as indicated by the AFM and TEM results. **(B)** Differences in adsorption onto HAP resulting in higher adsorption amounts for H8, and lower adsorption amounts for H3 compared to rpM179. The oligomer rings are oriented perpendicular to the surface for clarity in the schematic and because that orientation would promote interactions with the C-terminus. The orientation of rings onto the HAP surface is not known at the present time, however.

We were surprised to see significant increases in the adsorption amounts for SERK compared to rpM179 because of the removal of charged residues that have the potential for adsorption interactions. Also, it was remarkable to see that H3 had significantly lower amounts of adsorption compared to H2 and H8. The schematic in Figure 6 summarizes the trends in oligomer adsorption for H3, H8, and rpM179. To understand these results, we considered the effect of the protein charge since Supplementary Table S1 showed that there were differences in the calculated overall charge of the protein monomers at pH 8. A plot of adsorption coverage at 15.6 μg/mL as a function of the protein monomer charge at pH 8 in Supplementary Figure S9 showed a relatively weak correlation between the monomer charge and adsorption amount. Although there was a trend that Delta had a lower charge and lower adsorption amount compared to rpM179 while SEKR had the highest protein charge and a high adsorption amount, there were four proteins at moderate charge that had a wide range of adsorption amounts, ranging from 8% for H3 to 85% for H2. The charge of the protein monomer, therefore, may not be a very good predictor of the adsorption of the protein oligomer.

As discussed above, we observed that the removal of H and residues in the N-terminus, sequences that are known to be important in protein-protein interactions, led to changes in these interactions that resulted in smaller adsorbed oligomers compared to rpM179. We hypothesize that these changes in protein-protein interactions led to changes in the structure of the oligomer that in turn affected the adsorption interactions with HAP. For example, changes in the oligomer structure may have led to changes in the exposure of the C-terminus on the oligomer surface, modulating adsorption interactions with the HAP surface to either promote (H2, H8, SEKR) or inhibit (H3) adsorption interactions with HAP relative to rpM179. The change in oligomer quaternary structure might alter the surface charge of the oligomers, modifying their electrostatic interactions with the surface. Further studies of the structure of the oligomers adsorbed onto HAP using high resolution techniques will be necessary to develop a molecular level understanding of how the adsorption behavior is altered for the mechanistic probes compared to the wild-type protein.

### Amorphous calcium phosphate to hydroxyapatite phase transition

The mineralization of HAP involves the formation of ACP nanoparticles, their aggregation into larger structures, followed by their transformation to the thermodynamically stable phase, HAP (Tao et al., 2008; Habraken et al., 2013). Our mineralization studies showed that this transformation was inhibited by the protein adsorbates. It is well known that adsorbates can inhibit the ACP to HAP transformation (Posner et al., 1977), and adsorbed amelogenin has been observed to inhibit this phase transformation both *in vivo* (Beniash et al., 2009; Shin et al., 2020) and *in vitro* (Kwak et al., 2016; Tao et al., 2019). The induction time for the ACP to HAP transformation was significantly lower for Delta (rpM179 minus the C-terminus) compared to the wild-type protein. *In vivo* studies have shown that wild-type amelogenin is cleaved by MMP20 to form amelogenin without the C-terminus during the secretory stage of enamel formation. There is a gradient of wild-type protein during the secretory stage from high amounts in the freshly formed outer enamel to low amounts in the older, inner enamel (Uchida et al., 1991). Also, *in vivo* studies found that the older, inner enamel transforms to HAP before the outer, newer enamel (Beniash et al., 2009). Our *in vitro* results would predict that amelogenin regions within enamel that have more C-termini removed, the inner enamel region, would convert to HAP sooner and this is consistent with the *in vivo* results.

Although the mechanisms of the phase transformation from ACP to HAP have not been studied in detail in the presence of amelogenin, there are a number of previous studies of the transformation without protein. Several studies have shown that HAP nucleates onto the surface or within ACP particles and then the HAP nuclei grow (Tao et al., 2008; Xie et al., 2014; Lotsari et al., 2018). The ACP to HAP transformation, therefore, may be slowed by two mechanisms: 1) protein adsorption onto ACP that inhibits the interfacial nucleation of HAP and 2) protein adsorption onto HAP nuclei that inhibits the growth of HAP. Our AFM studies of protein adsorption onto HAP can provide direct insights into the inhibition of the phase transformation by the second mechanism. The adsorption of protein onto ACP, however, has not been studied previously or in this study because of the instability of ACP. Although the bulk structural unit of ACP has been found to be similar to that of HAP (Habraken et al., 2013), and trends in adsorption onto ACP and HAP might be similar, there is no data to confirm this at the present time.

Our previous study of single amino acid variants showed that an increase in the oligomer-HAP binding energy resulted in a longer ACP to HAP induction time (Tao et al., 2019). The variations in induction time between the mechanistic probe proteins, therefore, might relate to the binding energy of the proteins—the higher the binding energy, the greater the induction time. For mineralization studies at 200 μg/ml protein concentration, the AFM adsorption studies suggest that all of the proteins would have reached saturation adsorption and would form a complete oligomer monolayer in the first layer. The proteins with a lower binding energy, however, would have a higher degree of protein exchange with the solution at equilibrium, more easily allowing the incorporation of ions and promoting the nucleation of HAP onto ACP and the growth of the newly formed HAP nuclei. Proteins with a higher binding energy, on the other hand, would have a lower degree of protein exchange with the solution and would inhibit the flux of ions necessary for the ACP to HAP transformation as well as growth of HAP nuclei.

**FIGURE 7:**
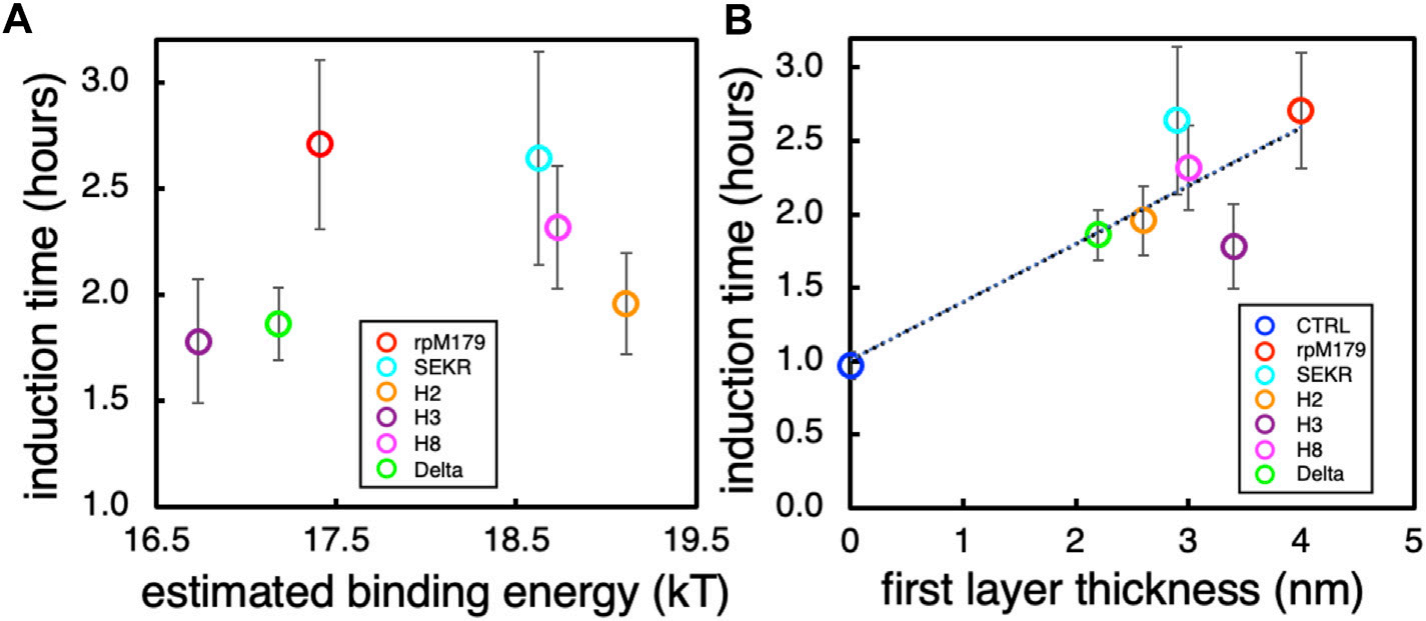
**(A)** The induction time for the ACP to HAP transformation as a function of the estimated binding energies obtained from Supplementary Figure S10. **(B)** The induction time as a function of the estimated thickness of the first layer of adsorbates obtained from the mean heights of the oligomers from Figure 2G.

Since we hypothesize that the induction times might be correlated with the oligomer-HAP binding energies and we have limited binding energy data in this study, we plotted the experimentally determined oligomer-HAP binding energies for H8, rpM179, and single amino acid variant proteins from our previous study (Tao et al., 2019) versus the equilibrium adsorption amounts at 15.6 μg/ml. Supplementary Figure S10 shows that there is a linear relationship between the oligomer-HAP binding energies that we have data for and the adsorption coverages at 15.6 μg/ml, suggesting that we can use the resulting equation of fit to estimate binding energies for the proteins in this study knowing their adsorption coverages at 15.6 μg/ml. Next, we plotted the induction times versus the estimated binding energies for proteins in this study to see if there is a relationship between the induction time and the oligomer-HAP binding energies (Figure 7A). This plot shows that the induction time for the ACP to HAP transformation increases with binding energy up to a point, but then decreases at higher binding energies above 18.5 kT. For example, H2 and H8 have shorter induction times than rpM179 even though they have higher estimated binding energies. This suggests that binding energy alone does not control the induction time and that other factors may be involved.

There were variations in the mean height of the oligomeric adsorbates for all of the proteins as shown in Figure 2G. At the protein concentration used for the mineralization experiments, 200 μg/ml, a complete monolayer of protein would be adsorbed in the first layer. Assuming that the thickness of the adsorbed protein layer can be approximated by the mean height of the oligomers adsorbed from Figure 2G, we plotted the induction times as a function of the first protein layer thickness as shown in Figure 7B. There was a general trend in increasing induction time with increasing protein layer thickness for most of the proteins. Even though H2 and H8 had higher binding energies than rpM179, they had lower protein thicknesses and shorter induction times. The protein thickness, therefore, may have a role in affecting the ACP to HAP phase transformation in combination with the binding energy. Since the transformation of ACP involves the nucleation of HAP and the growth of HAP from ions in the interfacial area, the adsorbates may block access of ions to the growing HAP nucleus. A thicker protein layer may be more effective in blocking ions compared to a thinner protein layer.

Further multivariate experimentation and analysis will be necessary to determine the relative importance of the oligomer binding energy and oligomer thickness to affect the ACP to HAP induction time. In addition, other factors may have roles in controlling the induction time. For example, the structure and orientation of the adsorbed oligomers may have an effect on controlling the diffusion of ions to the ACP surface. SEKR had a moderate protein thickness and a longer induction time than expected compared to the linear fit shown in Figure 7B. Previous solid state NMR (ssNMR) studies have shown that the N-terminus of rpM179 has a β-sheet structure when adsorbed onto the surface (Lu J.-X. et al., 2013; Arachchige et al., 2018). The removal of S16, E18, K24, and R31 may affect the secondary structure of amelogenin in the N-terminus resulting in a different oligomer structure and orientation on the surface that might prevent the diffusion of ions to the surface of ACP or the growing HAP nucleus even though the adsorbed layer is thinner. Studies of the secondary structure of the SEKR protein in the N-terminal region as well as the structure and orientation of the oligomer would be necessary to explain how the ACP to HAP transformation is inhibited by SEKR.

## Conclusion

Amelogenin is critical to the formation of enamel in teeth. The adsorption of amelogenin onto mineral surfaces has been found to be important in affecting the growth of calcium phosphates and the transformation of ACP to HAP both *in vivo* and *in vitro*. Our *in vitro* studies confirm that the C-terminus is the dominant domain promoting adsorption onto HAP surfaces and that histidines in the central region and SEKR residues in the N-terminus are primarily involved in controlling protein-protein interactions. The loss of the histidine and SEKR residues resulted in subtle changes in protein-protein interactions that affected the oligomer adsorbate sizes. We hypothesize that changes in these interactions also changed the oligomer quaternary structure to either promote (H2, H8, SEKR) or inhibit (H3) adsorption interactions relative to rpM179. Our studies confirmed the importance of amelogenin adsorption in affecting the ACP to HAP transformation and revealed new information on the mechanisms of this control. Mineralization studies found that the induction time for the ACP to HAP phase transformation was affected not only by the oligomer-HAP binding energy but also by the thickness of the adsorbed protein layer. The combination of mechanistic probes, *in situ* AFM monitoring, and mineralization kinetics studies have led to new insights into how the oligomer structure affects adsorption and calcium phosphate mineralization behavior. These studies reveal the importance of the amelogenin oligomer mesostructure on enamel mineralization *in vivo*.

## Data Availability

The raw data supporting the conclusions of this article will be made available by the authors, without undue reservation.
