# Supplementary material for: Changes in the C-terminal, N-terminal, and histidine regions of amelogenin reveal the role of oligomer quaternary structure on adsorption and hydroxyapatite mineralization
Source: Front Physiol. 2022 Nov 29;13:1034662. doi: 10.3389/fphys.2022.1034662 (PMC9746691; doi:10.3389/fphys.2022.1034662)
Supplement: Supplementary file 7 [file DataSheet1.pdf]

Supporting Information for:

Changes in the C-terminal, N-terminal, and Histidine Regions of Amelogenin Reveal the Role of Oligomer Quaternary Structure on Adsorption and Hydroxyapatite Mineralization

Jinhui Tao,[1] Emma Hanson,[1] Alice C. Dohnalkova,[1] Garry W. Buchko,[1,2] Biao Jin,[1] Wendy J. Shaw,[1*] Barbara J. Tarasevich[1]

[1]Pacific Northwest National Laboratory, Richland, WA 99354; [2]School of Molecular Biosciences, Washington State University, Pullman, WA 99164

*Corresponding author, wendy.shaw@pnnl.gov

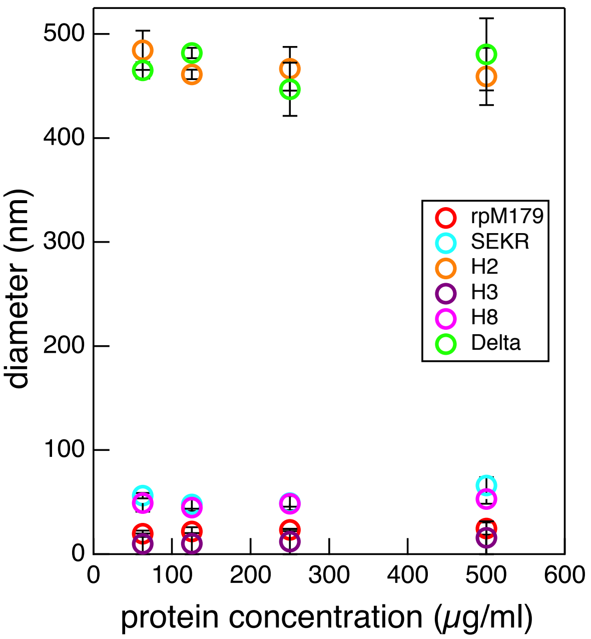

Figure S1. The mean diameters of nanospheres in pH 8, 25 mM Tris•HCl solutions at various protein concentrations as determined by DLS. The error bars are the standard deviation of the mean and may be smaller than the marker size. The average nanosphere diameters over the four concentrations are given in parentheses as follows: rpM179 (22 nm), SEKR (55 nm), H2 (430 nm), H3 (12 nm), H8 (47 nm) and Delta (470 nm).

Mechanistic probe solution nanosphere sizes. The nanosphere sizes of the mechanistic probes H2, H3, H8, Delta, SEKR, and wild type rpM179 (with sequences shown in Figure 1) in pH 8, Tris-buffered solutions were determined by dynamic light scattering (DLS). Figure S1

shows the average diameter of the structures as a function of protein concentration determined from the volume weighted NNLS size distribution. There was no significant change in size with concentration from 62.5 – 500 μg/mL, consistent with results from our previous studies on rpM179 and single amino acid variants (Tao et al., 2015a; Tao et al., 2019). The data show that all of the mechanistic probes and rpM179 aggregate to form nanospheres larger than a monomer, ranging in size from 12 nm in diameter for H3 to 470 nm in diameter for Delta. The nanosphere diameters varied as H3 (12 nm) < rpM179 (22 nm) < H8 (47 nm) ~ SEKR (55 nm) << H2 (430 nm) < Delta (470 nm). Previous studies also showed that Delta (rpM166) had larger nanosphere sizes than rpM179 (Moradian-Oldak et al., 2000). Since the protein adsorbates onto HAP (001) were oligomers, smaller than the nanospheres detected by DLS, we propose that the oligomers were present in solution but not detected by DLS.

AFM Imaging Experimental Details.

Some of this text is reproduced or modified from Tao et al. 2015 (Tao et al., 2015a). Micrometer-sized HAP single crystals with six lateral (100) faces were synthesized using the molten salt synthesis technique with potassium sulfate as the medium (Tas, 2001; Tao et al., 2015b). The morphology and surface indices of the HAP crystals were identified by SEM, AFM and XRD as reported in our previous publication (Tao et al., 2015b). To prepare HAP as a substrate for AFM imaging, the muscovite mica (diameter 9.9 mm, Ted Pella, Inc) was first fixed onto an AFM metal specimen disc (diameter 12 mm, Ted Pella, Inc). The mica was than freshly cleaved and treated by 10 µl of poly-L-lysine solution (0.1% w/v, Ted Pella, Inc) for 3 min, followed by thorough rinsing with water, and dried by a stream of nitrogen gas. Next, the poly-L-lysine functionalized mica was covered by 50 µl Tris-HCl buffer solution (25 mM, pH 8.0). Finally, the HAP crystals were bound onto the functionalized mica by putting 3 µl HAP aqueous suspension (0.1 wt%) on top of mica covered with the Tris-HCl buffer solution.

The sample was then transferred into an AFM equipped with a fluid cell. After stabilization for at least 10 min, about 400 µl of amelogenin solution with different concentrations buffered by Tris-HCl solution (25 mM, pH 8.0) was injected into the fluid cell while scanning over the HAP (100) faces. All in situ AFM images were collected in tapping mode with a Nanoscope 8 atomic force microscope (Digital Instruments J scanner, Bruker) at room temperature (22°C). The hybrid probes used consisted of silicon tips on silicon nitride cantilevers (HYDRA triangular lever, k=0.088 N/m, tip radius <10 nm; resonance frequency 75 kHz in air; Applied Nanostructures, Inc, www.appnano.com). The amplitude was set to be 20 nm (in fluid), and the signal-to-noise ratio was maintained above 10. The scanning speed was 1-2 Hz. The amplitude setpoint was carefully tuned to minimize the average loading force during the in situ imaging. Each concentration-dependent amelogenin adsorption experiment was repeated three times.

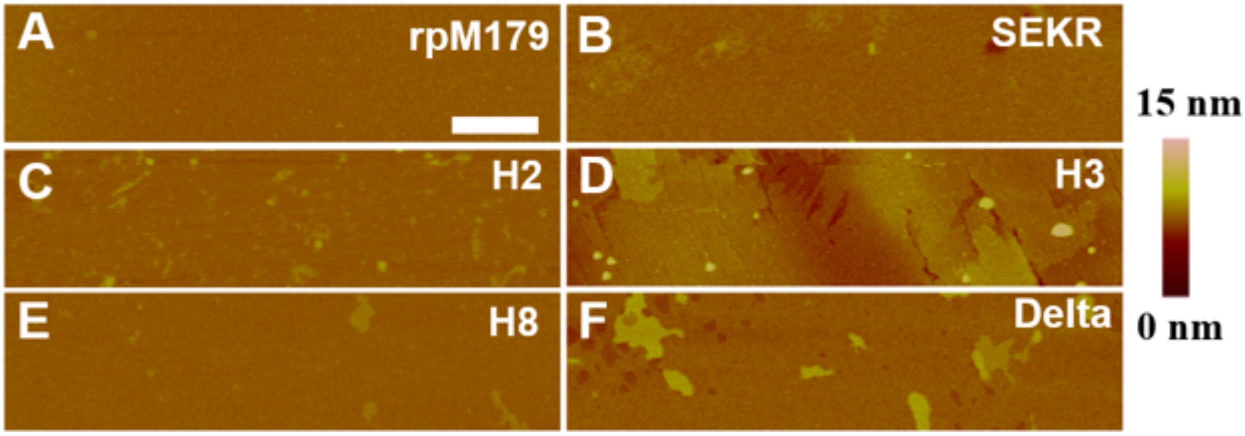

Figure S2. AFM images of the bare HAP (100) surface in buffer solution before injection of the protein solutions. The scale bar is 200 nm. All the micrographs have the same size.

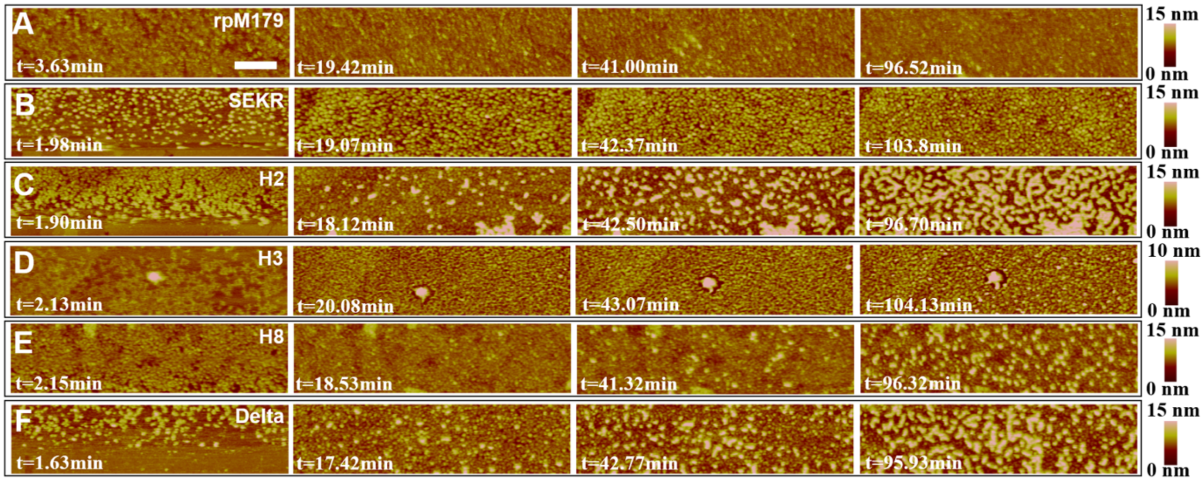

Figure S3. In situ AFM images of adsorbates from 125 µg/mL protein, 25 mM Tris•HCl, pH 8 solutions at various time periods and showing the formation of a second layer of adsorbates over the first layer of adsorbates. The zero point of time is defined as the time when protein solutions are injected into AFM liquid cell. The scale bar is 200 nm. All the micrographs have the same size. Figure S3A, panels 1 and 3 are taken from Tao et al. 2019 (Tao et al., 2019). The first panel shows first layer adsorption at partial coverage for SEKR, H2, and Delta and nearly full coverage for rpM179, H3, and H8. The second, third, and fourth panels show second layer adsorption. The adsorption amounts are very low for rpM179 and H3 and are higher for SEKR, H2, H8, and Delta.

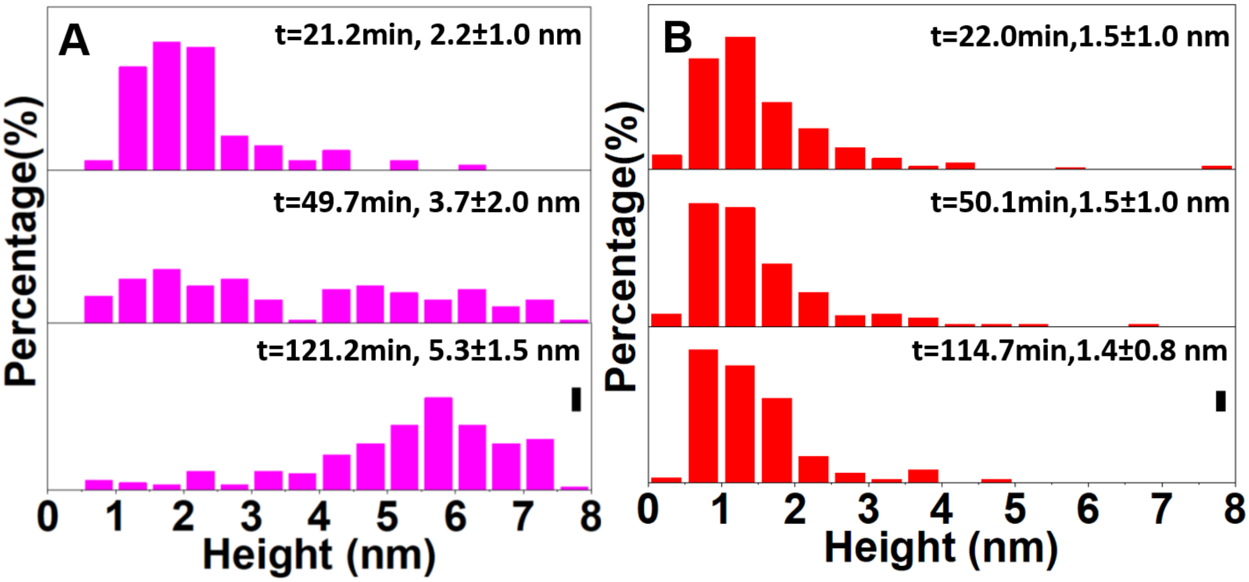

Figure S4. AFM oligomer height distributions over time for the second adsorbed layer of H8 (A) and rpM179 (B) at 125 μg/mL protein concentration.

Hill analysis of the equilibrium adsorption coverages.

Most of this text is reproduced from the Supplementary Information in Tao et al. 2019 (Tao et al., 2019). The experimentally determined dependencies of equilibrium adsorption coverage on protein concentration as shown in Figure S2 were used to determine the binding constants and binding free energies for both the amelogenin oligomer-HAP and oligomer-oligomer interactions (Table 1) for rpM179, Delta and H8 as demonstrated previously. The adsorption process of amelogenin onto HAP (100) can be described by the Hill analysis (Hill, 1910; Stefan and Le Novere, 2013) as:

$$n\mathrm{Amel} + \mathrm{B} \overset{\mathrm{K}_a}{\leftrightarrow} (n\mathrm{Amel} \bullet \mathrm{B}) \quad \text{(E1)}$$

where n is the number of amelogenin oligomers (or, in some cases, nanospheres), Amel is the free amelogenin oligomer, B is the available binding site on the surface, Amel·B is the adsorbed amelogenin oligomer, and K_a is the equilibrium binding constant. The analysis uses the number of adsorbates, n, regardless of the adsorbate size.

The coverage at the equilibrium state based on Equation E1 is:

$$\theta = \frac{[\mathrm{Amel}]^n}{\mathrm{K}_a^{-1} + [\mathrm{Amel}]^n} \quad \text{(E2)}$$

where θ represents the fraction of the surface covered by protein, [Amel] is the protein concentration in solution given in M (mol/L), and K_a has units of M^{-n}. θ is experimentally determined as the areal coverage of protein by AFM.

The Hill equation is derived from a linear formulation of equation E2:

$$\ln\left(\frac{\theta}{1-\theta}\right) = n\ln[\mathrm{Amel}] + \ln\mathrm{K}_a \quad \text{(E3)}$$

where n is the Hill coefficient describing cooperativity. The Hill coefficient provides a way to quantify a cooperative effect during the binding processes. It is a measure of how protein already bound to the surface effects the binding of additional protein. A coefficient of one indicates completely independent binding, regardless of how many additional ligands are already bound. A Hill coefficient greater than one indicates positive cooperativity, with protein-protein interactions contributing to the total amount of protein binding. The protein-protein interactions described by n are interactions lateral to the protein that is already adsorbed. An analysis was performed using the Langmuir equation, a reduced form of the Hill equation where n =1, however, this analysis did not result in good fits. The oligomer equilibrium coverages including repetitions from several experiments for each protein concentration were plotted according to Equation E4 and least squares analysis was done to obtain a linear fit. The slope (n) and intercept (K_a) were obtained from the linear fit and the oligomer-HAP binding free energy was calculated from equation E4. For solution amelogenin at a "standard" concentration, normally 1 M, the standard binding free energy (ΔG) per mole of amelogenin is:

$$\Delta G = -\frac{1}{n} k_B T \ln K_a \qquad (E4)$$

where k_B is Boltzmann's constant and T is the absolute temperature.

The standard deviation of ΔG in Table 1 is calculated by:

$$\frac{\sigma(\Delta G)}{\overline{\Delta G}} = \sqrt{\left(\frac{\sigma(lnKa)}{\overline{lnKa}}\right)^2 + \left(\frac{\sigma(n)}{\bar{n}}\right)^2} \qquad (E5)$$

where $\sigma(\Delta G)$, $\sigma(lnKa)$, and $\sigma(n)$ are the standard deviations for the binding free energy, *lnKa* and *n*. $\overline{\Delta G}$, $\overline{lnKa}$, and $\bar{n}$ are the average value for the binding free energy, *lnKa* (intercept) and *n* (slope), respectively. The values for $\sigma(lnKa)$, and $\sigma(n)$ were determined from the least squares analysis of the linear fits.

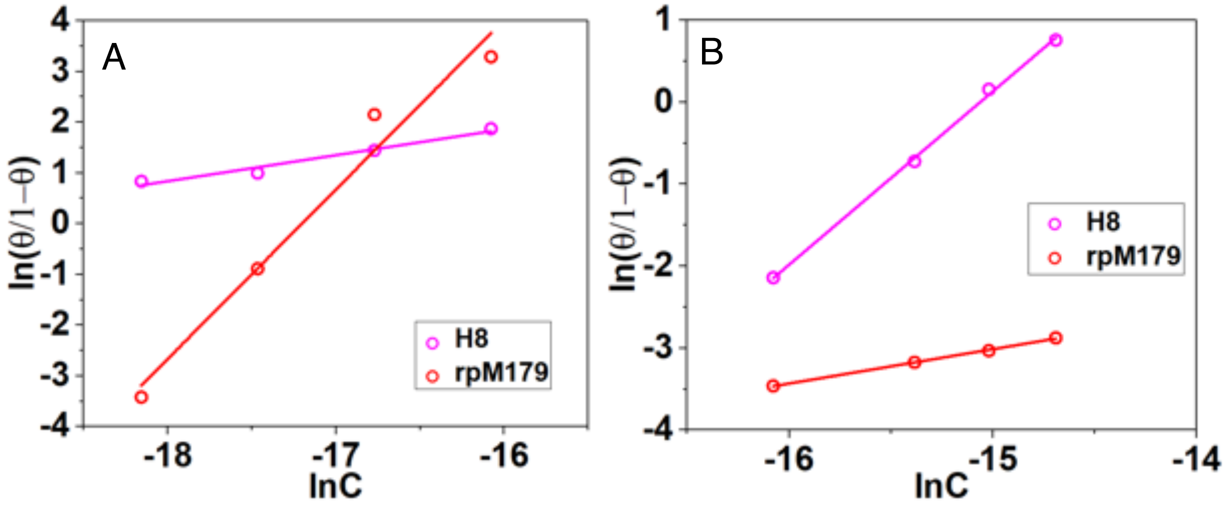

Figure S5. Hill plots of ln(θ/1-θ) versus lnC where θ is the equilibrium adsorption coverage and C is the protein concentration in solution for: (A) first layer adsorption and (B) second layer adsorption. The slopes of the fitted lines are n and the intercepts are ln K_a. The data used for rpM179 includes data points from this study combined with data points from Tao et al. 2019 (Tao et al., 2019).

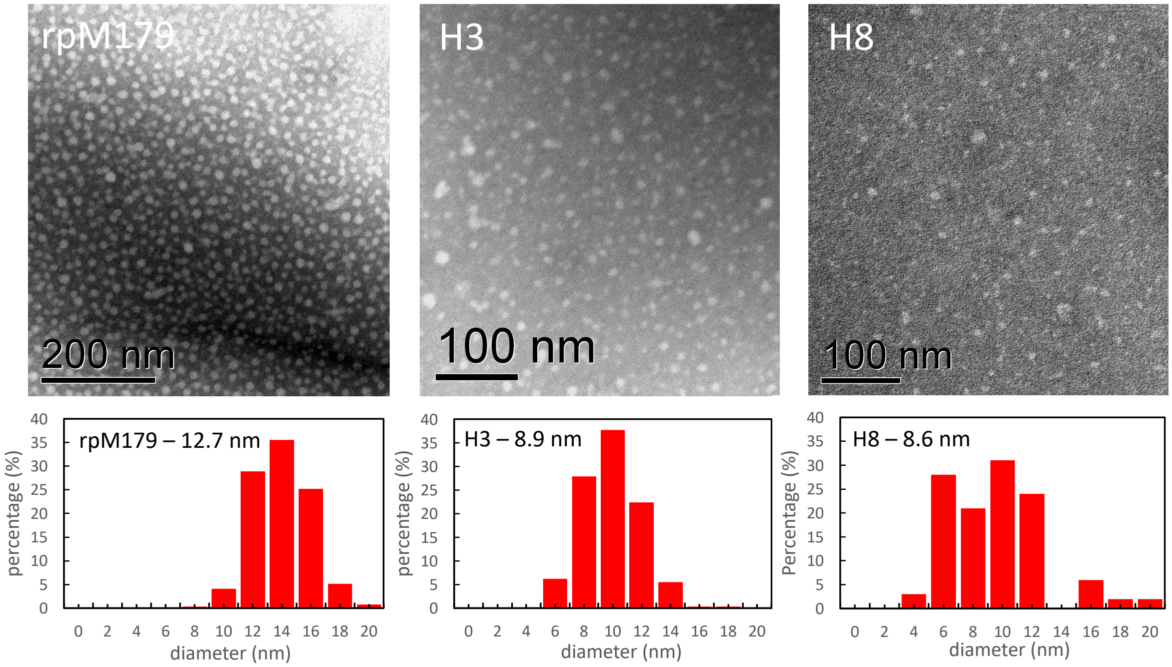

Figure S6. (A) Images of amelogenin oligomers adsorbed onto TEM grids. (B) Size distributions of oligomer diameters determined by measurement of at least 100 oligomers. The mean diameter size is shown in the label.

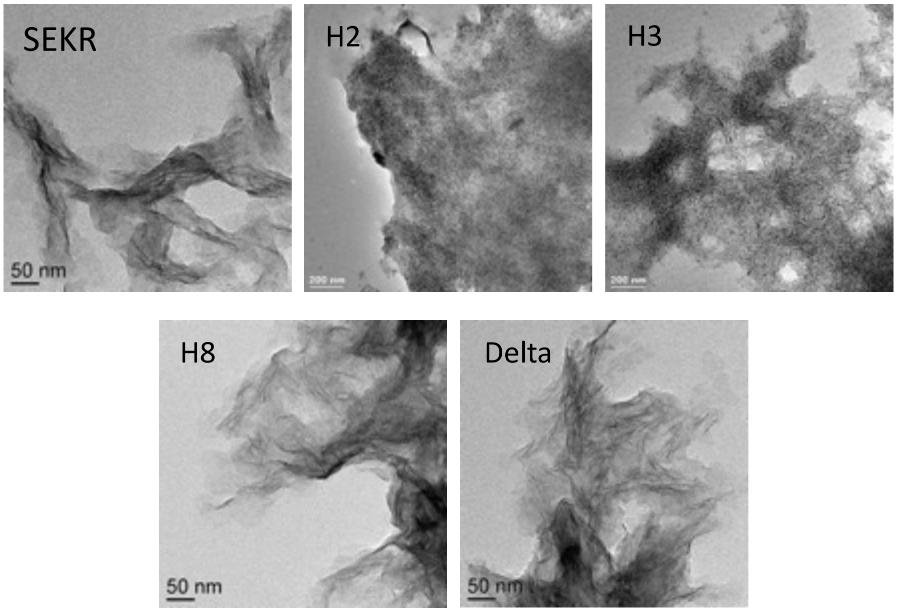

Figure S7. TEM images of precipitates resulting from the mineralization of calcium phosphate after 24 hours in the presence of 200 µg/mL mechanistic probe proteins SEKR, H2, H3, H8, and Delta at 22°C.

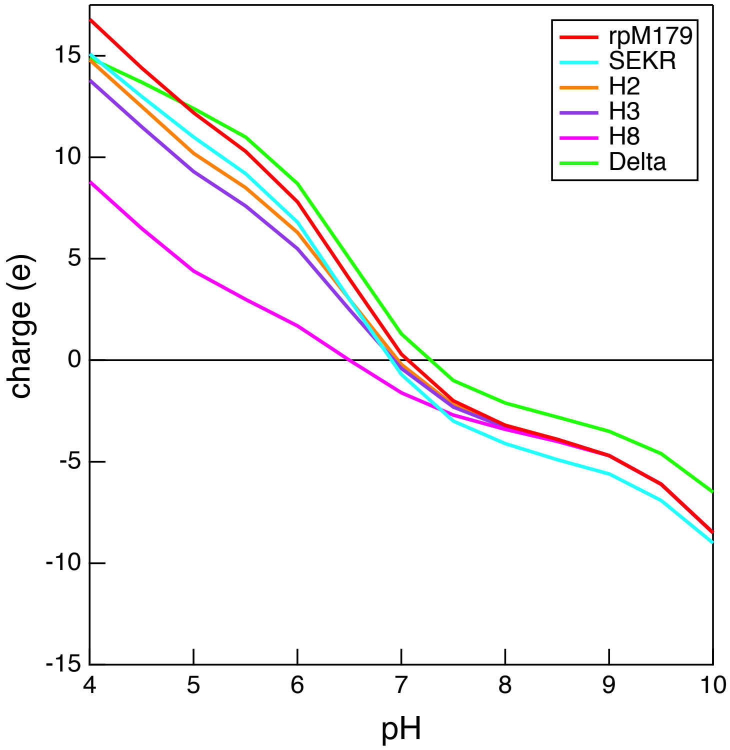

Figure S8. The overall charge in elementary charge units, e, of rpM179 and the mechanistic probes as a function of pH calculated from http://protcalc.sourceforge.net/.

Table S1. Charge at pH 8, isoelectric point, aliphatic index, and grand average hydropathy of wild type and mechanistic probe variant proteins.

**Protein**	**Charge at pH 8 (e)**[1]	**Isoelectric point (pH)**[1]	**Grand average hydropathy**[2]	**Aliphatic Index**[2]
Delta	-2.1	7.23	-0.620	67.53
H2	-3.2	6.96	-0.668	65.73
rpM179	-3.2	7.05	-0.696	65.00
H3	-3.3	6.91	-0.654	66.10
H8	-3.4	6.52	-0.580	68.02
SEKR	-4.1	6.88	-0.640	66.48

[1] http://protcalc.sourceforge.net/
[2] https://web.expasy.org/protparam/

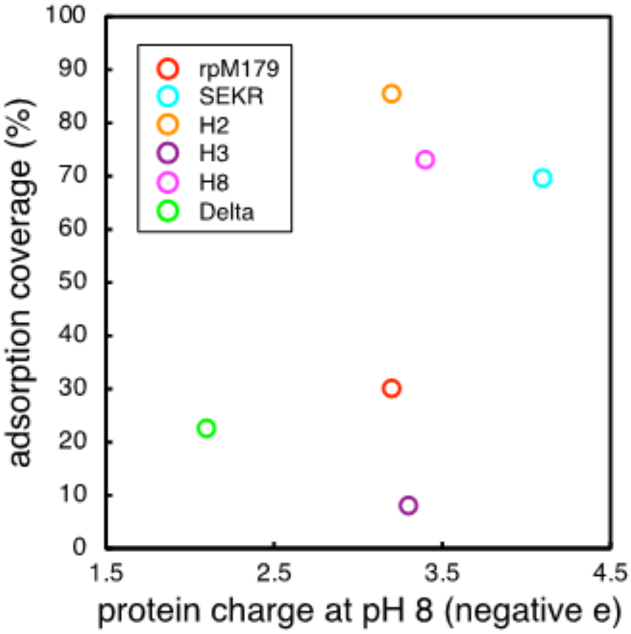

Figure S9. The equilibrium adsorption amounts of proteins at 15.6 μg/mL as a function of the overall negative protein monomer charge at pH 8.

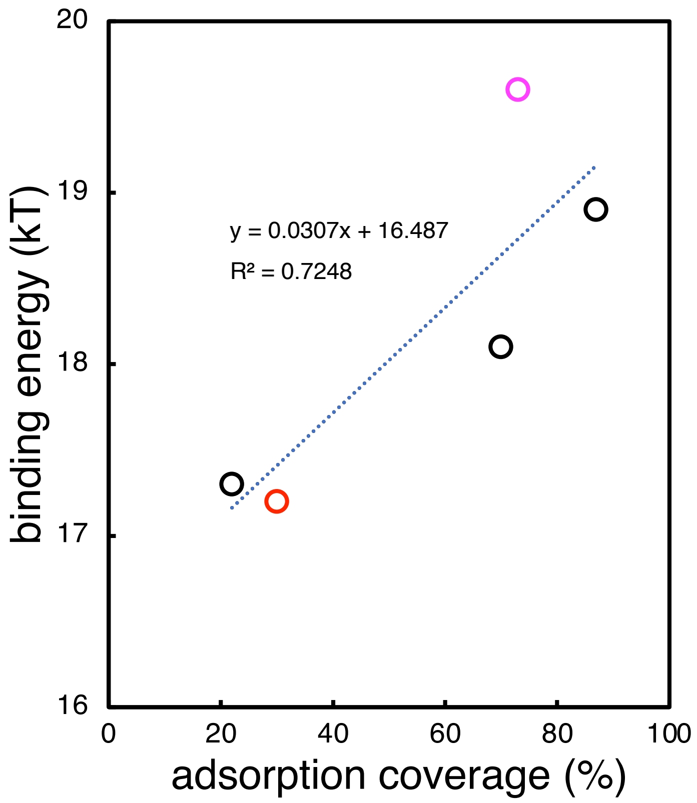

Figure S10. The oligomer-HAP binding energy for versus the equilibrium adsorption coverage at 15.6 µg/mL protein concentration for H8 (magenta circle) from this study and rpM179 (red circle), T21I, P41T, and P71T from Tao et al. 2019 (Tao et al., 2019) showing the equation and R^2 for the linear fit.

List of Movies:

The movies show AFM images of protein at various concentrations in ppm (equivalent to µg/mL) adsorbed onto HAP (100) over time. The rpM179 movies, M1 and M7, are from Tao et al. 2019 (Tao et al., 2019).

Video 1 - M1 M179 15.6 ppm
Video 2 - M2 SEKR 15.6 ppm
Video 3 - M3 H2 15.6 ppm
Video 4 - M4 H3 15.6 ppm
Video 5 - M5 H8 15.6 ppm
Video 6 - M6 Delta 15.6 ppm
Video 7 - M7 M179 125 ppm
Video 8 - M8 SEKR 125 ppm
Video 9 - M9 H2 125 ppm
Video 10 - M10 H3 125 ppm
Video 11 - M11 H8 125 ppm
Video 12 - M12 Delta 125 ppm
Video 13 - M13 H8 8 ppm
Video 14 - M14 H8 31 ppm
Video 15 - M15 H8 63 ppm

References

Hill, A.V. (1910). The heat produced in contracture and muscular tone. *J. Physiol.-London* 40**,** 389-403.

Moradian-Oldak, J., Paine, M.L., Lei, Y.P., Fincham, A.G., and Snead, M.L. (2000). Self-assembly properties of recombinant engineered amelogenin proteins analyzed by dynamic light scattering and atomic force microscopy. *J.Struct. Biol.* 131**,** 27-37.

Stefan, M.I., and Le Novere, N. (2013). Cooperative binding. *Plos Comp. Biol.y* 9. doi: ARTN e1003106 10.1371/journal.pcbi.1003106.

Tao, J., Buchko, G.W., Shaw, W.J., De Yoreo, J.J., and Tarasevich, B.J. (2015a). Sequence-defined energetic shifts control the disassembly kinetics and microstructure of amelogenin adsorbed onto hydroxyapatite (100). *Langmuir* 31**,** 10451-10460. doi: 10.1021/acs.langmuir.5b02549.

Tao, J., Battle, K.C., Pan, H., Salter, E.A., Chien, Y.C., Wierzbicki, A., et al. (2015b). Energetic basis for the molecular-scale organization of bone. *Proc. Natl. Acad. Sci. U S A* 112**,** 326-331. doi: 10.1073/pnas.1404481112.

Tao, J., Shin, Y., Jayasinha, R., Buchko, G.W., Burton, S.D., Dohnalkova, A.C., et al. (2019). The energetic basis for hydroxyapatite mineralization by amelogenin variants provides insights into the origin of amelogenesis imperfecta. *Proc. Natl. Acad. Sci. USA.* 116**,** 13867-13872. doi: 10.1073/pnas.1815654116.

Tas, A.C. (2001). Molten salt synthesis of calcium hydroxyapatite whiskers. *J. Am. Ceram. Soc.* 84**,** 295.
